# Circulating MicroRNA-150 Serum Levels Predict Survival in Patients with Critical Illness and Sepsis

**DOI:** 10.1371/journal.pone.0054612

**Published:** 2013-01-23

**Authors:** Christoph Roderburg, Mark Luedde, David Vargas Cardenas, Mihael Vucur, David Scholten, Norbert Frey, Alexander Koch, Christian Trautwein, Frank Tacke, Tom Luedde

**Affiliations:** 1 Department of Medicine III, University Hospital Rheinisch-Westfaelische Technische Hochschule (RWTH) Aachen, Aachen, Germany; 2 Department of Cardiology and Angiology, University of Kiel, Kiel, Germany; University of São Paulo, Brazil

## Abstract

**Background and Aims:**

Down-regulation of miR-150 was recently linked to inflammation and bacterial infection. Furthermore, reduced serum levels of miR-150 were reported from a small cohort of patients with sepsis. We thus aimed at evaluating the diagnostic and prognostic value of miR-150 serum levels in patients with critically illness and sepsis.

**Methods:**

miR-150 serum levels were analyzed in a cohort of 223 critically ill patients of which 138 fulfilled sepsis criteria and compared to 76 healthy controls. Results were correlated with clinical data and extensive sets of routine and experimental biomarkers.

**Results:**

Measurements of miR-150 serum concentrations revealed only slightly reduced miR-150 serum levels in critically ill patients compared to healthy controls. Furthermore miR-150 levels did not significantly differ in critically ill patients with our without sepsis, indicating that miR-150 serum levels are not suitable for diagnostic establishment of sepsis. However, serum levels of miR-150 correlated with hepatic or renal dysfunction. Low miR-150 serum levels were associated with an unfavorable prognosis of patients, since low miR-150 serum levels predicted mortality with high diagnostic accuracy compared with established clinical scores and biomarkers.

**Conclusion:**

Reduced miR-150 serum concentrations are associated with an unfavorable outcome in patients with critical illness, independent of the presence of sepsis. Besides a possible pathogenic role of miR-150 in critical illness, our study indicates a potential use of circulating miRNAs as a prognostic rather than diagnostic marker in critically ill patients.

## Introduction

MicroRNAs (miRNAs) represent small RNAs of 22 nucleotides in length, that do not withhold the sequential information to transcribe for proteins, but function as critical regulators of gene expression in eukaryotes [1], [2]. miRNAs are involved in highly regulated processes such as cell injury, proliferation or carcinogenesis [3], [4], [5]. In inflammation and immunity, miRNAs were demonstrated to regulate central elements of the innate and adaptive immune response such as antigen presentation and T cell receptor signaling [6]. Furthermore, alterations of miRNA expression were found in chronic autoimmune diseases such as ulcerative colitis or Crohn’s disease [7], suggesting a role for miRNAs in immune-mediated diseases. It is thus tempting to speculate that miRNAs are dysregulated in conditions of systemic inflammation, especially sepsis, as well.

Besides their functional involvement in gene expression, the regulation of miRNAs in body fluids has raised growing attention with regards to the potential role as biomarkers in human disease [8]. However, at present little is known about miRNAs deregulated in the serum of patients with critical illness and sepsis. Recently, it was demonstrated that serum levels of two miRNAs – miR-146a and miR-223– were significantly reduced in septic patients compared to SIRS patients and healthy controls [9]. Interestingly, these respective miRNAs are also involved in the pathogenesis of inflammation and bacterial infection [6]. Thus, besides serving as biomarkers, alterations in serum miRNA-levels may also reflect pathophysiological processes occurring in sepsis. It is therefore likely that, besides miR-146 and miR-223, other miRNAs associated with regulatory processes in a setting of sepsis can serve as potential biomarkers.

miR-150 was recently identified as a key regulator of immune cell differentiation and activation [10], [11]. miR-150 is preferentially expressed in mature, resting T and B cells but not in their progenitors as maturation of B-cell and T-cell lead to a dramatic down-regulation of miR-150. Ectopic expression of miR-150 in hematopoietic stem cell progenitors reduces mature B-cell levels in the peripheral blood as well as in solid immune-organs like spleen and lymph nodes by preventing the transition from pro-B to pre-B cell, thus providing a molecular basis for the impaired immune responses observed in miR-150^−/−^mice [10], [11]. Moreover, LPS injections into healthy humans resulted in reduced expression levels of miR-150 in leukocytes [12]. Interestingly, miR-150 serum levels were found to be reduced in a small cohort of 16 patients with sepsis [13]. Based on these observations, we analysed in a large, well-defined cohort of patients the diagnostic and prognostic value of miR-150 serum levels in critical illness and/or sepsis.

## Materials and Methods

### Study Design and Patient Characteristics

In the present study, we enrolled 223 patients (140 male, 83 female, with a median age of 63 years; range 18 to 89 years), that were consecutively admitted to the General Internal Medicine intensive care unit (ICU) at the University Hospital Aachen (Table 1). Patients who were expected to have a short-term (<72 h) intensive care treatment due to post-interventional observation or acute intoxication were not included into this study [14]. The clinical course of patients was observed in a follow-up period of three years by directly contacting the patients, the patients’ relatives or their primary care physician. Patients who met the criteria proposed by the American College of Chest Physicians and the Society of Critical Care Medicine Consensus Conference Committee for severe sepsis and septic shock were categorized as sepsis patients, the others as non-sepsis patients [15], [16], [17]. As a control population we analyzed 76 healthy blood donors (47 male, 29 female, median age 33 years, range 18–67) with normal values for blood counts, C-reactive protein and liver enzymes.

**Table 1:** Baseline patient characteristics.

Parameter	all patients	Non-sepsis	sepsis	P-value
Number	223	85	138	n.a.
Sex (male/female)	140/83	55/30	90/48	n.s.
Age median (range) [years]	63 (18–89)	61 (18–85)	64 (20–89)	n.s.
APACHE-II score median (range)	17 (2–43)	15 (2–43)	18.0 (3–40)	n.s
SAPS2 score median (range)	42.5 (0–79)	41 (13–72)	43 (0–79)	n.s.
ICU days median (range)	7 (1–83)	5 (1–45)	10 (1–83)	<0.001
Death during ICU (%)	23.6%	14.6%	28.7%	0.021
Death during ICU or follow-up (%)	43.9%	28.1%	52.9%	<0.001
Ventilation time median (range) [h]	129 (0.5–1363)	45 (0–820)	207 (0.5–1363)	0.005
Body mass index (BMI)	26.08 (16.6–86.5)	26 (16.6–53.3)	26.12 (18.3–86.5)	n.s.
Creatinine	1.3 (0–15)	1 (0.3–15)	1.5 (0–10.7)	n.s.
Albumin	27.0 (15.2- 52.2)	29.2 (15.2–52.2)	25.7 (17.3–51.0)	n.s.
WBC median (range) [×10^3^/µl]	12.15 (0.1–67.4)	11.4 (1.8–27.7)	12.7 (0.1–67.4)	n.s.
CRP median (range) [mg/dl]	94.0 (<5–230)	17 (5–230)	165 (<5–230)	<0.001
Procalcitonin median (range) [µg/l]	0.7 (0–180.6)	0.2 (0.05–100)	2.3 (0–180.6)	<0.001
Interleukin-6 median (range) [pg/ml]	110 (0–83000)	63 (4–83000)	220 (0–28000)	<0.001
Tumor necrosis factor median [pg/ml]	19 (4.9–140)	16.5 (8.0–100)	23.5 (4.9–140)	0.009
miR-150 median (range) [rel. ex.]	17.5 (0.04–2150.35)	22.94 (0.08–1853.14	14.52 (0.04–2150.35)	n.s.

APACHE, Acute Physiology and Chronic Health Evaluation; CRP, C-reactive protein; ICU, intensive care unit; SAPS, simplified acute physiology score; WBC, white blood cell count.

Patients were included into the study upon providing written informed consens and the ethics committees approved this consent procedure. The study protocol was approved by the local ethics committee and conducted in accordance with the ethical standards laid down in the Declaration of Helsinki (ethics committee of the University Hospital Aachen, RWTH-University, Aachen, Germany, reference number EK 150/06).

### Characteristics of Sepsis and Non-sepsis Patients

138 of the 223 patients included in this study conformed to the criteria of bacterial sepsis at the time point of admission to the ICU (Table 1) [15], [16], [17]. Pneumonia represented the most prominent source of infection (Table 2). In non-sepsis patients cardiopulmonary diseases (myocardial infarction, pulmonary embolism, and cardiac pulmonary edema), decompensated liver cirrhosis or other critical conditions represented the predominant etiologies, which did not differ in age or sex from sepsis patients. Sepsis patients were more often in need of mechanical ventilation in longer terms as compared to the non-sepsis patients’ cohort (Table 1). In sepsis patients significantly higher levels of routinely used biomarkers of inflammation (C-reactive protein, procalcitonin, white blood cell count) were found (Table 1). Both groups did not differ in Acute Physiology and Chronic Health Evaluation (APACHE) II, and Simplified Acute Physiology Score (SAPS)2 score, vasopressor demand, or laboratory parameters indicating liver or renal dysfunction (Table 1).

**Table 2:** Disease etiology of the study population.

	sepsis	non-sepsis
	n = 138	n = 85
**Sepsis critical illness**Source of infection n (%)		
Pulmonary	74 (53.6%)	
Abdominal	28 (20.3%)	
Urogenital	3 (2.2%)	
Other	32 (23.1%)	
**Non-sepsis critical illness**n (%)		
cardiopulmonary disease		31 (36.5%)
decompensated liver cirrhosis		12 (14.1%)
non-sepsis other		43 (50.6%)

### Cell Culture and Stimulation

The monocyte cell line U937 cells were cultured in RPMI medium with 10% fetal bovine serum, 300 mg/L L-glutamine and penicillin/streptomycin. For differentiation, cells were seeded on 6-well-plates and stimulated with 0,1 µg/ml PMA for 24 hours and further left untreated or stimulated for 48 h with 1 µg/ml LPS, respectively.

### miRNA-Isolation from U937 Cells

Total RNA was purified from U937 cells using Qiazol reagent (Qiagen, Hilden, Germany) and miRNeasy Mini kit (Qiagen) according to the manufacturer’s protocol, and was resuspended in suitable amounts of H_2_O.

### miRNA Isolation from Serum

400 µl serum was spiked with miScript miRNA mimic SV40 (Qiagen 2 µM, 1 µl/100 µl serum) for sample normalization. 800 µl phenol (Qiazol) and 200 µl chloroform were added to the sample and mixed vigorously for 15 sec followed by an incubation at room temperature for 10 min. Samples were centrifuged for 30 min at 12,000 g until complete phase separation. The aqueous phase, containing total RNA, was precipitated with 500 µl 100% isopropanol and 2 µl glycogen (Fermentas, St. Leonroth, Germany) overnight at −20°C. After centrifugation at 4°C for 15 min (12,000 g) the pellets were washed once with 70% ethanol. Precipitated RNA was resuspended in 30 µl RNase free water (Ambion, Austin, TX). To asses the quantity and quality of RNA, the samples were measured with a NanoDrop spectrophotometer (NanoDrop), and a smallRNA assay for Agilent´s Bioanalyzer was performed (Agilent Technologies, Böblingen, Germany) [18], [19], [20].

### Quantitative Real-time PCR

Quantitative real-time PCR was performed as recently described [18], [19], [20]. In detail, the quantity and quality of the RNA was determined using a nanodrop spectrophotometer (NanoDrop, Wilmington, DE). Total RNA (1 µg) was used to synthesize cDNA utilizing miScript Reverse Transcriptase Kit (Qiagen) according to the manufacturer’s protocol, and was resuspended in suitable amounts of H_2_O. cDNA samples (2 µl) were used for quantitative real-time PCR in a total volume of 25 µl using the miScript SYBR Green PCR Kit (Qiagen) and miRNA specific primers (Qiagen) on a qPCR machine (Applied Biosystems 7300 Sequence Detection System, Applied Biosystems, Foster City, CA). All real-time PCR reactions were performed in duplicates. Data, using the 2?-ΔΔCT method (ΔCT target Gene - ΔCT control gene), were presented as relative gene expression. Data were generated and analyzed using the SDS 2.3 and RQ manager 1.2 software packages.

### Statistical Analysis

Data are displayed as median and range considering the skewed distribution of most parameters. Differences between two groups were assessed by Mann-Whitney- U-test and multiple comparisons between more than two groups have been conducted by Kruskal-Wallis- ANOVA and Mann-Whitney-U-test for post hoc analysis. Box plot graphics illustrate comparisons between subgroups and display a statistical summary of the median, quartiles, range and extreme values. The whiskers extend from the minimum to the maximum value excluding outside and far out values which are displayed as separate points. An outside value (indicated by an open circle) was defined as a value that is smaller than the lower quartile minus 1.5-times the interquartile range, or larger than the upper quartile plus 1.5-times the interquartile range. A far out value was defined as a value that is smaller than the lower quartile minus three times the interquartile range, or larger than the upper quartile plus three times the interquartile range. All values, including “outliers”, have been included for statistical analyses. Correlations between variables have been analysed using the Spearman correlation test, and values of P<0.05 were considered statistically significant. Kaplan Meier curves were plotted to display the impact on survival. Receiver operating characteristic (ROC) curve analysis and the derived area under the curve (AUC) statistic provide a global and standardized appreciation of the accuracy of a marker or a composite score for predicting an event. ROC curves were generated by plotting sensitivity against 1-specificity. All statistical analyses were performed with SPSS version 12.0 (SPSS, Chicago, IL, USA).

## Results

### Concordant Regulation of miR-150 Levels in Immune Cells and Supernatant

**Figure 1:**
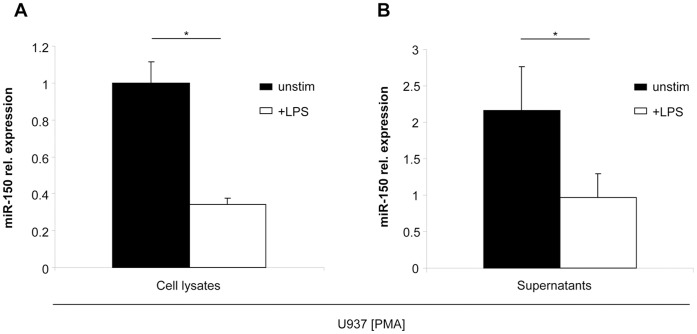
miR-150 is down-regulated upon stimulation with LPS. U937 cells were stimulated with PMA into a monocytic differentiation. miR-150 expression levels in response to stimulation with LPS for 48 h were measured by qPCR within cells (A) and cell supernatant (B).

Based on the previously proposed functional relation between miR-150 levels in serum and peripheral immune cells [12], [13], we first tested how stimulation of monocytic cells with bacterial lipopolysaccharide (LPS) affects intra- and extracellular miR-150 levels in cell culture. Stimulation of cultured U937 cells with LPS led to a significant down-regulation of miR-150 in these cells (Figure 1 A). This specific regulation in U937 cells was associated with lower levels of this distinct miRNA in the supernatant of stimulated cells when compared to non-stimulated cells (Figure 1 B), indicating a link between intra- and extracellular miR-150 levels upon immune cell activation. Of note, levels of miR-571, which was recently demonstrated to be unaltered LPS-treated U937 cells [19], were unaltered in the supernatants of LPS treated U937 cells, when compared to non-stimulated cells (Supporting Information S1).

### miR-150 Serum Levels in Critically ill Patients and Healthy Controls

**Figure 2:**
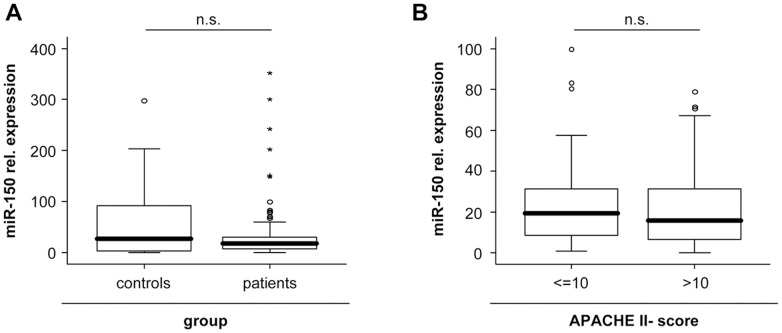
Serum miR-150 levels of critically ill patients at ICU admission. (A) Serum miR-150 levels at admission to the intensive care unit were analyzed by qPCR and revealed a trend toward reduced miR-150 concentrations in critically ill patients (n = 223) as compared with healthy controls (n = 76). (B) Serum miR-150 concentrations at admission to the ICU were not significantly changed in critically ill patients with high initial Acute Physiology and Chronic Health Evaluation (APACHE) II scores (>10) in comparison to patients with low APACHE-II-scores (</ = 10). Box plot are displayed, where the bold line indicates the median per group, the box represents 50% of the values, and horizontal lines show minimum and maximum values of the calculated non-outlier values; asterisks and open circles indicate outlier values.

Based on a previous small pilot study suggesting a dysregulation of miR-150 serum levels in sepsis patients [12], [13], we measured concentrations of miR-150 in sera of 223 patients at admission to the ICU as well as in 76 healthy volunteers. Unexpectedly, miR-150 serum levels were not different in our large cohort of patients with critical illness compared to healthy controls (P = 0.232; Figure 2 A). Moreover, when we analyzed serum levels in patients with severe disease indicated by APACHE-II-scores >10, no differences to patients with lower APACHE-II-scores (<10) were observed (Figure 2 B). Recently, a correlation of miR-150 serum concentration with the SOFA sequential organ failure assessment (SOFA)-score was described in a small cohort of patients with septic disease. However, no correlation between miR-150 and any of the common clinical disease severity scores (SOFA; APACHE-II, SAPS2) was found (Table 3).

**Table 3:** Correlations of miR-150 serum concentrations at ICU admission with other laboratory markers.

	ICU patients	
Parameter	R	p
***Markers of liver function***		
cholinesterase	0.039	n.s.
protein	−0.039	n.s.
albumin	−0.034	n.s.
INR	0.009	n.s.
***Markers of inflammation***		
C-reative protein	−0.061	n.s.
procalcitonin	0.038	n.s.
IL-6	0.086	n.s.
IL-10	0.136	n.s.
TNF-alpha	−0.036	n.s.
***Markers of renal function***		
creatinine	−0.284	<0.001
cystatin C	−0.241	0.005
cystatin C GFR	0.287	0.007
urea	−0.303	<0.001
***Others variables***		
lactate	−0.227	0.001
NT-proCNP	−0.228	0.005
glucose	−0.147	0.03
neutrophile counts	−0.241	0.008
platelets	−0.153	0.024
***Clinical scoring***		
APACHE-II	−0.086	n.s.
SOFA	−0.053	n.s.
SAPS2	−0.102	n.s.

r, correlation coefficient; p, p-value; r and p-values by Spearman rank correlation; INR, intenational normalized ratio; IL-6, Interleukin-6; IL-10, Interleukin-10; TNF, tumour necrosis factor; APACHE-II, Acute Physiology and Chronic Health Evaluation II; SOFA, Sequential Organ Failure Assement Score.

In contrast, miR-150 levels closely correlated with different single laboratory markers of organ failure. As such, miR-150 serum levels showed a strong and significant correlation with decreased renal function with respect to glomerular filtration rate of cystatin C (r = 0.287, P = 0.007), creatinine (r = −0.284, P<0.001) and urea (r = −0.303, P<0.001) serum concentrations (Table 3). Finally, miR-150 serum concentrations strongly correlated with serum lactate levels, thus underlining a role of miR-150 as a marker for organ dysfunction in critically ill patients.

The metabolic status of patients was shown to influence the outcome of critically ill patients [21]. Since miR-150 was recently found to be down-regulated in the adipose tissue of patients with obesity and non-alcoholic steatohepatitis [22] we analyzed potential correlations between miR-150 serum levels and the presence of obesity or type 2 diabetes. These analyses revealed no significant differences in circulating miR-150 between patients with or without type 2 diabetes mellitus (Supporting Information S2A) and those with obesity or normal body weight, respectively (Supporting Information S2B). Of note, all of these results remained unchanged when raw Ct values prior to SV-40 normalization were used, demonstrating the robustness of these results.

### miR-150 Serum Levels are Unchanged in Patients with Sepsis

**Figure 3:**
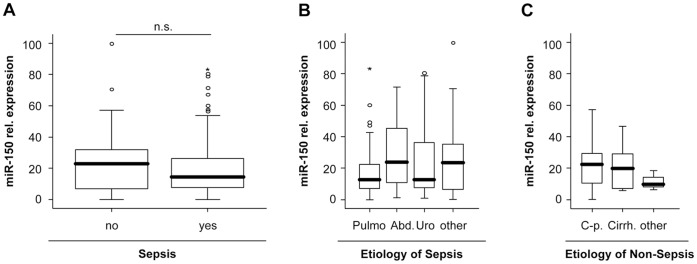
Serum miR-150 concentrations are unaltered in sepsis. (A) miR-150 serum levels were unchanged in patients that fulfilled sepsis criteria (n = 157) compared to patients with non-septic etiology of critical illness. (B) miR-150 serum did not vary between the different sources of septic diseases (C) miR-150 serum did not vary between the different etiologies of non-septic diseases.

As stated before, our large, well defined cohort of ICU patients featured both patients fulfilling sepsis criteria (n = 138) and patients without sepsis (n = 85). miR-150 serum levels were previously shown to be downregulated in a small cohort of 16 patients with sepsis. Thus, we further investigated the impact of inflammation and sepsis on circulating miR-150 levels in our cohort. This analysis revealed no significant differences in circulating miR-150 levels between these septic and non-septic patients (Figure 3 A; Supporting Information S3). The lack of an association of circulating miR-150 with the presence of sepsis was further substantiated by correlation analyses revealing that serum miR-150 levels were not significantly correlated to established markers of systemic inflammation and bacterial infection such as C-reactive protein (CRP), procalcitonin (PCT), interleukin-6 (IL-6), interleukin-10 (IL-10) or tumor necrosis factor (TNF) in critically ill patients (Table 3). Additionally, we performed subgroup analyses in order to figure out whether miR-150 serum levels, despite being unchanged in the whole collective of septic patients, might be altered in distinct etiologies of diseases. These analyses revealed a tendency towards higher miR-150 serum levels in the group of patients with abdominal sepsis. However, differences did not reach statistical significance (Figure 3 B, C). Collectively, these data show that, in contrast to previous findings in a small cohort of 16 septic patients [13], median miR-150 serum levels were not significantly altered in septic patients compared to non-septic critically ill patients or healthy controls, indicating that it might not be a valuable biomarker for the diagnosis of sepsis.

### miR-150 Serum Concentrations Predict Survival in Critically Ill Patients

**Figure 4:**
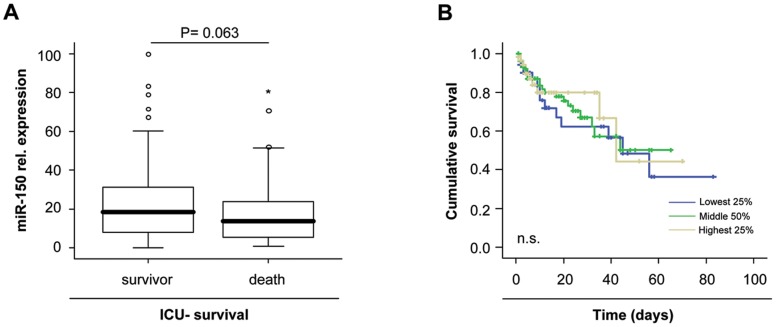
Prediction of ICU mortality by miR-150 serum concentrations. (A) Patients that die during the course of ICU treatment had a tendency towards lower miR-150 serum levels on admittance to ICU than survivors (P = 0.063, U-test). Box plot are displayed, where the bold line indicates the median per group, the box represents 50% of the values, and horizontal lines show minimum and maximum values of the calculated non-outlier values. (B) Kaplan-Meier survival curves of ICU patients are displayed, showing that patients with miR-150 levels within the lowest quartile of all patients displayed the lowest short-term survival at the ICU. Significances are given in the figure.

Despite the fact that median miR-150 serum levels did not significantly vary between septic and non-septic critically ill patients, we tested whether they might be useful in predicting mortality in critically ill patients. We therefore compared miR-150 levels in patients that died during the ICU treatment to those from survivors. Interestingly, patients that survived showed a tendency towards higher miR-150 serum concentrations compared to patients that died during ICU treatment, although statistical significance was not reached (P = 0.063; Figure 4 A). Similarly, Kaplan Meyer curve analyses revealed that patients with high miR-150 levels showed a trend towards a better ICU prognosis (Figure 4 B).

**Figure 5:**
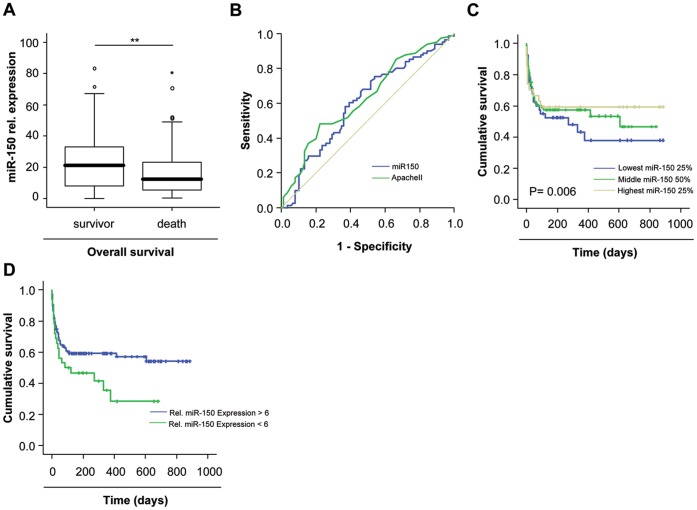
Low miR-150 serum concentrations are associated with an impaired prognosis of critically ill patients. (A) Long-term surviving patients had higher serum miR-150 levels on admittance to ICU (P = 0.009, U-test) compared to patients that died during long term follow up which displayed significantly lower miR-150 levels. Box plot are displayed, where the bold line indicates the median per group, the box represents 50% of the values, and horizontal lines show minimum and maximum values of the calculated non-outlier values. (B) ROC curve analyses revealed a high prognostic accuracy of miR-150 for overall survival compared to the APACHE-II-score. (C) Kaplan-Meier curve analysis of ICU patients revealing that patients with miR-150 concentrations within the lower quartile had an increased overall mortality as compared to patients with miR-150 serum concentrations of highest quartile. P-values for the Cox regression analysis are given in the figure. (D) Kaplan-Meier survival curves of ICU patients showed that patients with low miR-150 concentrations had an increased overall mortality in the long-term follow-up compared to patients with higher miR-150 levels. ** P<0.01.

In our cohort of critically ill patients, 23.6% died on the ICU while additional 20.3% of patients died after initially successful discharge from ICU. Strikingly, patients with long-term survival demonstrated significantly higher miR-150 levels upon ICU-admission than those that died during follow-up (P = 0.009; Figure 5 A). In line, ROC curve analysis showed that miR-150 as a single parameter displayed a similar AUC as the multifactorial APACHE-II-score (AUC_miR-150_∶0.636 vs. AUC_APACHEII_: 0.534; Figure 5 B). Furthermore, when compared to other single parameters, routinely used by many intensivists to predict patients prognosis, miR-150 demonstrated the best predictive capacity for long-term survival in critically ill patients (AUC_CRP_: 0.593; AUC_Creatinine_: 0.558; AUC_bilirubin_: 0.497; AUC_leucocytes_: 0.467; AUC_urea_: 0.618; AUC_lactate_: 0.581; Supporting Information S4).

To determine the prognostic value of miR-150 measurements in critically ill patients we performed Cox regression and Kaplan Meyer curve analyses. Upon Cox regression, miR-150 were closely associated with prognosis (P = 0.006). Kaplan-Meier curves displayed that miR-150 levels within the highest quartile indicated a significantly favorable prognosis (Figure 5 C). As shown in Figure 5 D, a cut-off value for miR-150 serum levels of 6 relative units allowed to clearly discriminate between long-term survivors and patients that will die in the long term. Finally, the prognostic power of miR-150 serum levels for long time patients mortality even increased during the follow-up period (Table 4). Collectively, our data indicate that measurement of miR-150 levels upon admission to the ICU might have a valuable role in the assessment of a critically ill patient’s short-term and long-term prognosis.

**Table 4:** Mortality of critically ill patients in relation to their miR-150 serum concentrations at ICU admission to the ICU.

	circulating miR-150		
	lowest 25%	middle 50%	highest 25%
**30-days mortality**	31.4%	29.3%	25.6%
**60-days mortality**	42.9%	46.2%	41.2%
**90-days mortality**	49.0%	48.4%	45.5%
**180-days mortality**	58.8%	54.3%	51.6%
**365-days mortality**	75.0%	69.6%	55.2%

## Discussion

miR-150 has recently been proposed as a novel marker for sepsis in a small cohort of 16 critically ill patients [13]. It was suggested that low miR-150 serum levels identify septic disease in these patients and that miR-150 levels correlate with classical prognosis scores such as SOFA or established markers for sepsis and inflammation such as TNF, IL-10 or IL-18 [13]. However, the limited number of investigated patients, as well as the missing evaluation of confounding factors such as age, gender, body mass index and disease etiology might been regarded as limitations of this small pilot study.

In the present study we demonstrate that miR-150 serum levels appear slightly reduced in patients with critical illness; however, despite the very high number of tested patients, this regulation failed statistical significance. These differences to the previously published results might very likely be related to the size and characteristics of the patient cohorts analyzed in the different studies. We report data from a large consecutively recruited cohort that covered a broad spectrum of critically ill patients with regard to severity of illness as reflected by APACHE II and SOFA score. Moreover, the experimental procedures were slightly different in the different studies: while we used spiked-in RNA (SV40) for normalization of miR-150 levels, which is presently considered as “golden standard” for miRNA analysis from the serum [23], Vasilescu et al., in contrary, used an internal miRNA, namely miR-192, for normalization [13]. Of note, our results remained unchanged when raw C_t_-values, prior to any normalization, were used for analysis. Thus, in contrast to prior observations, our data indicate that miR-150 serum levels are not deregulated in critically ill patients at the time-point of admission to the ICU and do not reflect the presence of sepsis in these patients.

miRNAs have recently been associated with the pathogenesis of inflammation and sepsis [6]. Down-regulation of miR-150 was described in leukocytes of human volunteers upon treatment with LPS [12]. Furthermore, miR-150 was suggested to regulate the differentiation and the activation state of immune cells by alterations of c-Myb signaling and expression levels of CXCR4, both representing direct targets of miR-150 [24], [25]. Most importantly, miR-150^−/−^ mice demonstrated significant changes in their immune cell populations and responses towards different inflammatory stimuli [26]. In line to these previous results we demonstrate here a significant down-regulation of miR-150 in U937 cells upon stimulation with LPS. Remarkably, this intracellular regulation was reflected by lower miR-150 levels in supernatants of these cells. However, these previous *in vitro* data as well as our own *in vitro* data could not been reproduced in an in vivo setting as in our large collective of ICU patients, miR-150 concentrations did not differ in patients that fulfilled sepsis criteria compared to those with other cause for critical illness. Furthermore, no correlations between miR-150 serum levels and markers of inflammation and bacterial infection, such as CRP or PCT could be detected. In contrary, we detected a clear correlation of miR-150 serum levels with serum concentrations of lactate and other classical indicators of hepatic and renal organ failure. More importantly, in our analysis, low levels of miR-150 were significantly associated with an unfavorable long-term prognosis and miR-150 predicted patients’ outcome with a higher accuracy than routinely used parameters such as CRP, procalcitonin, creatinine, bilirubine or serum lactate (Supporting Information S3). This important finding might be explained by the fact that complex pathological processes such as organ failure and patients survival might be better reflected by analysis of miRNAs which integrate both proinflammatory and anti-inflammatory signals in critical illness, than by measurement of single proteins alone. Thus, our data for the first time highlight a currently unknown role of miR-150 in processes determining outcome of critically ill patients on an ICU. However, the exact functional mechanisms how circulating miRNAs and especially circulating miR-150 determines patientś prognosis in critical illness are presently unclear. It was recently suggested that miR-150 is selectively packaged into microvesicles and actively secreted by human blood cells [27]. Such microvesicles allow delivering of miRNAs into cells, where they regulate expression of their respective target genes [28], [29]. In this setting, lower levels of circulating miR-150 might lead to a derepression of genes such as CXCR4 and c-Myb, which have both been liked with the activation of immune response of the one hand and patientś prognosis on the other.

Despite enormous advances in diagnostic modalities, triaging patients at the Emergency Room for relocation to the ICU or guiding therapeutic decisions within the first week of ICU treatment represent major challenges in the treatment of critically ill patients [30]. Remarkably, low miR-150- serum concentrations upon admission to the medical ICU were an unfavorable indicator for ICU survival as well as for overall survival (Figures 4 and 5). Patients that died during follow up displayed significantly lower miR-150 concentrations than survivors. Of note, the association with survival was independent from other parameters by multivariate regression analysis, thus further underscoring the robustness of our findings.

In summary, our study is the first to demonstrate the prognostic value of miRNA measurements in the serum of ICU patients. miR-150 serum concentrations upon admission were closely associated with ICU survival as well as long-term survival, and low miR-150 levels indicated an unfavourable prognosis. Our data provide evidence for a potential role of serum miRNAs concentrations and especially of serum miR-150 levels as a novel prognostic tool in critically ill patients. Such data might help to improve prognostic assessments in critically ill patients upon admission to the ICU and should provoke further basic research on the functional involvement of miR-150 in conditions of critical illness and systemic inflammation e.g by using miR-150^−/−^ mouse in animal models of sepsis.

## Supporting Information

Supporting Information S1 U937 cells were stimulated with PMA into a monocytic differentiation. miR-571 expression levels in response to stimulation with LPS for 48 h were measured by qPCR within cell supernatant.
(PDF)

Supporting Information S2 Serum miR-150 levels are independent of type 2 diabetes and obesity (A) Serum miR-150 concentrations are unaltered in critically ill patients with or without diabetes. (B) Serum miR-150 concentrations are unaltered in critically ill patients with or without obesity (defined as body-mass index >30 kg/m^2^ at ICU admission). Box plot are displayed, where the bold line indicates the median per group, the box represents 50% of the values, and horizontal lines show minimum and maximum values of the calculated non-outlier values; asterisks and open circles indicate outlier values.
(PDF)

Supporting Information S3 Serum miR-150 concentrations are unaltered in sepsis. miR-150 serum levels were unchanged in patients that fulfilled sepsis criteria compared to patients with non-septic etiology of critical illness or controls.
(PDF)

Supporting Information S4 ROC curve analyses revealed a high prognostic accuracy of miR-150 serum levels for overall survival compared to well established markers of sepsis or organ failure.
(PDF)
