# Supplementary figures and images for: Circulating MicroRNA-150 Serum Levels Predict Survival in Patients with Critical Illness and Sepsis
Source: PLoS One. 2013 Jan 23;8(1):e54612. doi: 10.1371/journal.pone.0054612 (PMC3555785; doi:10.1371/journal.pone.0054612)

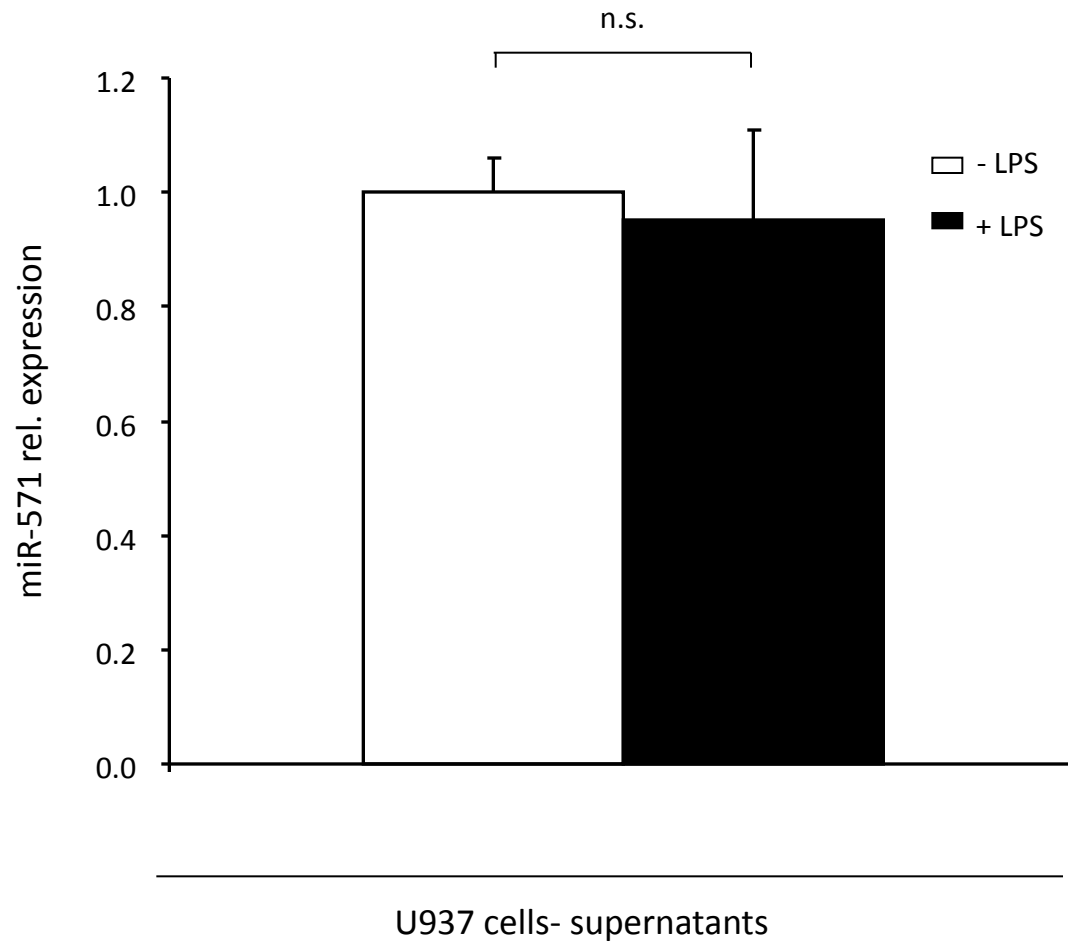

Supplement: Supporting Information S1 — U937 cells were stimulated with PMA into a monocytic differentiation. miR-571 expression levels in response to stimulation with LPS for 48 h were measured by qPCR within cell supernatant. (PDF) [file pone.0054612.s001.pdf]

**A**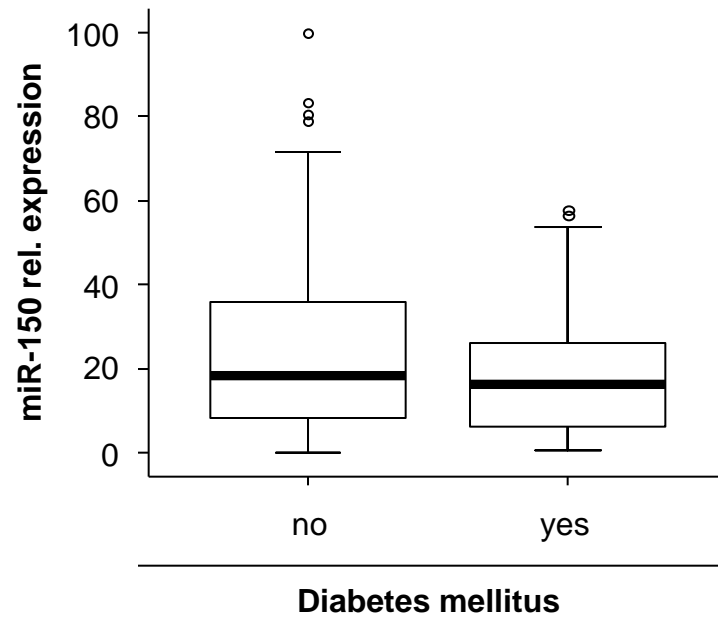**B**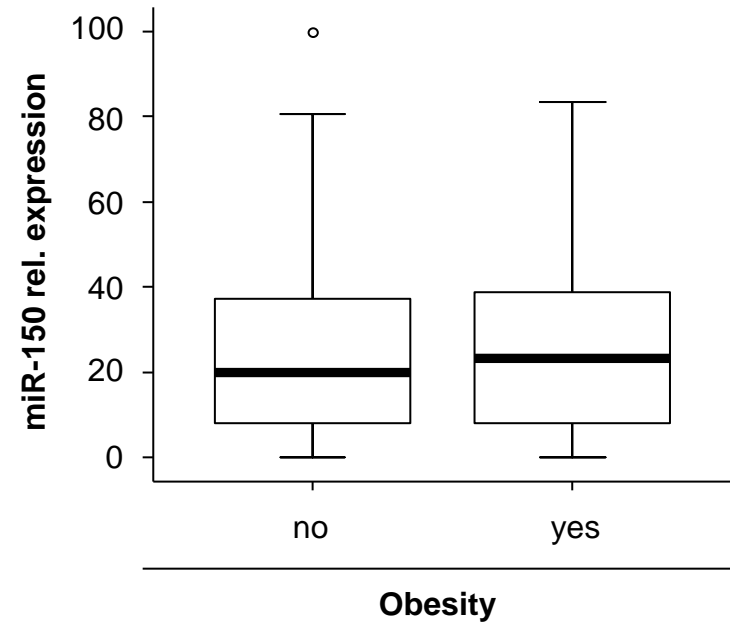

Supplement: Supporting Information S2 — Serum miR-150 levels are independent of type 2 diabetes and obesity (A) Serum miR-150 concentrations are unaltered in critically ill patients with or without diabetes. (B) Serum miR-150 concentrations are unaltered in critically ill patients with or without obesity (defined as body-mass index >30 kg/m2 at ICU admission). Box plot are displayed, where the bold line indicates the median per group, the box represents 50% of the values, and horizontal lines show minimum and maximum values of the calculated non-outlier values; asterisks and open circles indicate outlier values. (PDF) [file pone.0054612.s002.pdf]

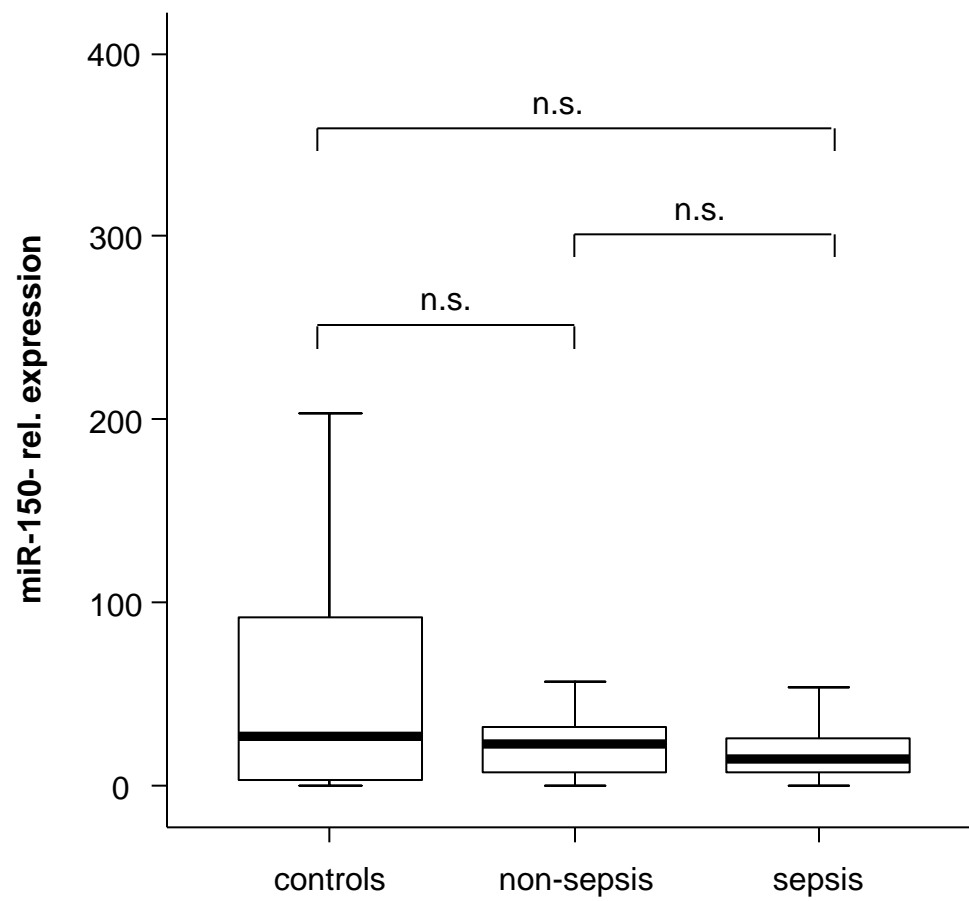

Supplement: Supporting Information S3 — Serum miR-150 concentrations are unaltered in sepsis. miR-150 serum levels were unchanged in patients that fulfilled sepsis criteria compared to patients with non-septic etiology of critical illness or controls. (PDF) [file pone.0054612.s003.pdf]

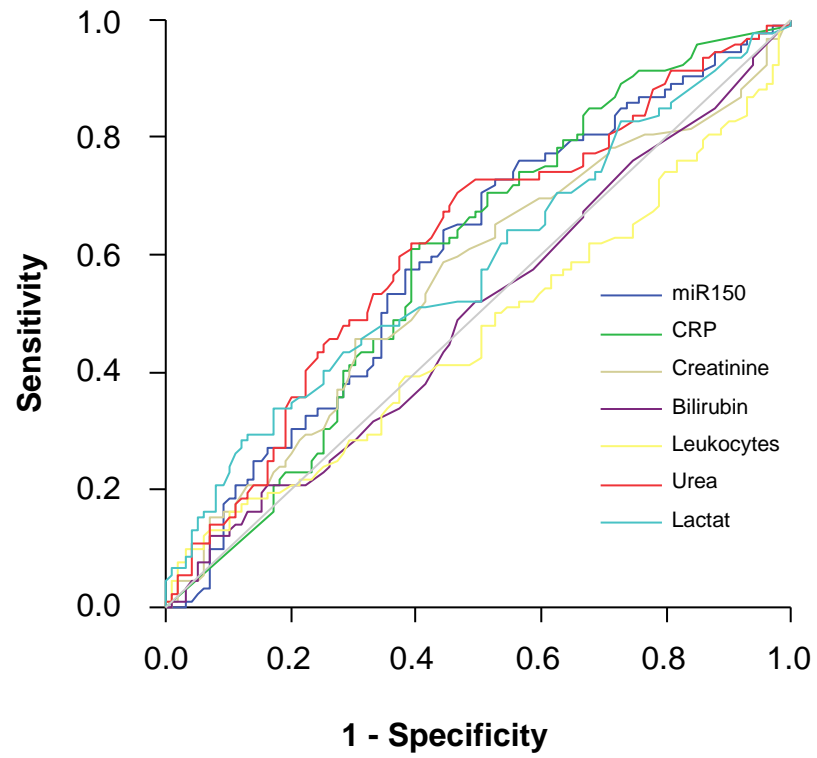

Supplement: Supporting Information S4 — ROC curve analyses revealed a high prognostic accuracy of miR-150 serum levels for overall survival compared to well established markers of sepsis or organ failure. (PDF) [file pone.0054612.s004.pdf]
